# Mesenchymal stem cell-derived exosomes as cell-free therapeutics for sensorineural hearing loss

**DOI:** 10.17305/bb.2025.11517

**Published:** 2025-03-06

**Authors:** Maria Perde-Schrepler, Ioana Brie, Alma Maniu

**Affiliations:** 1 Iuliu Hatieganu, University of Medicine and Pharmacy, Department of Oto-Rhyno-Laryngology, Cluj-Napoca, Romania; 2 Ion Chiricuta, Institute of Oncology, Cluj-Napoca, Romania

**Keywords:** Sensorineural hearing loss, SNHL, exosomes, inner ear, mesenchymal stem cells, MSCs.

## Abstract

Sensorineural hearing loss (SNHL) can result from various factors, including ototoxic drugs (such as aminoglycosides and chemotherapeutic agents), prolonged exposure to intense sound, and autoimmune or genetic disorders. In adult mammals, the loss of sensory cells in the cochlea is irreversible due to their lack of regenerative capacity. Current treatment options include hearing aids for mild to moderate hearing loss, which rely on residual hearing, and cochlear implants for severe cases, which provide limited auditory recovery while leading to the loss of any remaining natural hearing. Stem cell therapies, particularly those involving mesenchymal stem cells (MSCs), are being increasingly explored in regenerative medicine. MSCs are multipotent cells capable of differentiating into mesodermal lineage cells and possess immunomodulatory and regenerative properties, making them potential candidates for SNHL treatment. However, their administration carries risks, including unwanted differentiation, immune system activation, and potential tumorigenic effects. Exosomes, extracellular vesicles in the nanometer size range, are secreted by most eukaryotic cells. These vesicles, which have a double lipid membrane and contain genomic and proteomic material, play a crucial role in intercellular communication. Exosomes derived from MSCs exhibit similar biological functions to their parent cells but with significantly lower risks, as they do not trigger immune responses or pose oncological concerns. This paper aims to review current knowledge on the use of MSCs and MSC-derived exosomes for inner ear sensory cell regeneration and explore their potential for clinical applications.

## Introduction

**Figure 1.**
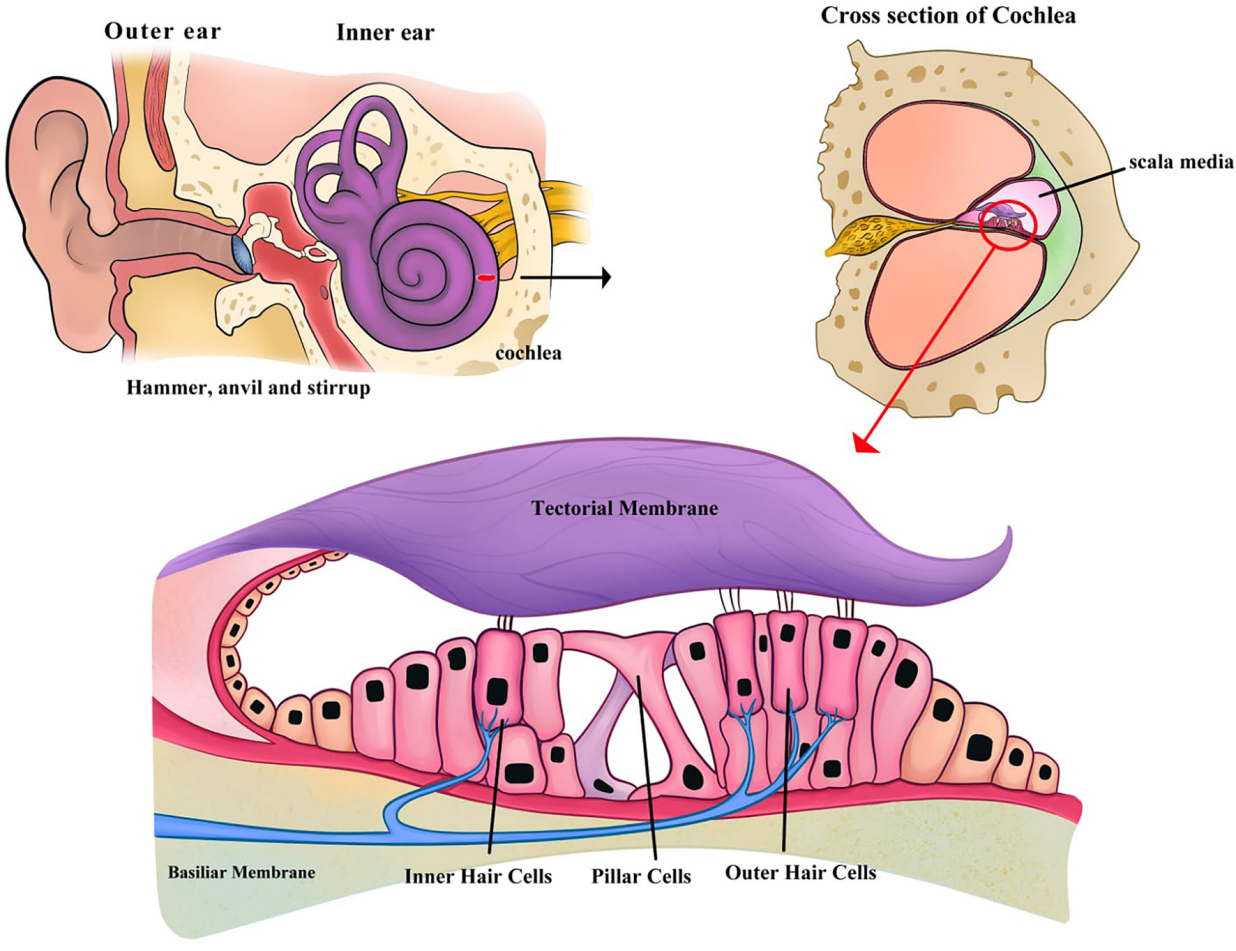
The organ of Corti, located in the scala media—an endolymph—filled cavity inside the cochlea.

Sensorineural hearing loss (SNHL) is the most common type of hearing impairment [1]. The World Health Organization (WHO) estimates that approximately 6% of the global population experiences some degree of hearing loss [2]. SNHL affects communication, speech, and cognition, significantly impacting social life, education, employment, and the economy. Hearing is primarily facilitated by the organ of Corti, located in the scala media—an endolymph-filled cavity inside the cochlea. This organ contains approximately 15,000 inner and outer hair cells (HCs) arranged in a specific pattern: a single row of inner HCs and three rows of outer HC, separated by supporting cells (SCs) [3] (Figure 1). The stereocilia and kinocilia of the HCs, in contact with the tectorial membrane, convert sound vibrations into electrical signals, which are transmitted as action potentials along the cochlear nerve and auditory pathways to the brain [4]. During embryonic development, between embryonic days E13 and E15, mammalian cochlear sensory cells lose their regenerative capacity. Once these cells are destroyed, their loss is irreversible beyond this time point [5–8]. As a result, hearing loss in adult mammals is permanent. Currently, there are no fully effective treatment options for SNHL [9]. The gold standard for treatment is the cochlear implant (CI), a device in which electrodes are surgically implanted into the patient’s cochlea to bypass damaged HCs and directly stimulate auditory neurons. Although CIs significantly improve speech perception and quality of life [10], they have several drawbacks, including difficulty hearing in noisy environments, challenges in music perception, and the risk of further damage to the already compromised inner ear during surgery [11–13]. Glucocorticoids are commonly used to treat inner ear conditions due to their anti-inflammatory effects, but their efficacy is limited. Moreover, long-term corticosteroid use is often associated with serious side effects [14–16]. Other therapeutic approaches include growth factors, such as epidermal growth factor (EGF), brain-derived neurotrophic factor (BDNF), and insulin-like growth factor 1 (IGF-1), which have shown moderately positive outcomes [17–19]. Gene therapy has also been explored, particularly through the transfection of Atoh1—a transcription factor crucial for neural and inner ear HC development—or the otoferlin (OTOF) gene in patients with hereditary OTOF mutations causing SNHL. While promising results have been observed, clinical trials remain limited due to significant adverse effects, as well as challenges in formulation and targeted delivery [20–25].

Mesenchymal stem cells (MSCs) are multipotent cells isolated from various organs and tissues. They can differentiate into multiple mesodermal cell types and play a crucial role in immunomodulation, making them a promising option for treating damaged cochlear sensory epithelium by replacing lost HCs or neurons. Additionally, MSCs serve as an excellent source of exosomes (MSC-Exo)—membrane-bound vesicles carrying bioactive molecules, such as peptides, proteins, and RNA. These exosomes facilitate intercellular communication, exerting biological effects similar to their parent cells while posing lower risks. The development of an effective treatment for SNHL that fully restores the inner ear’s structure and function depends on understanding the molecular mechanisms underlying its limited regenerative capacity. In recent years, significant efforts have been made to explore new strategies to prevent inner ear damage or stimulate the regeneration of neurosensory cells. This review aims to summarize and critically analyze the existing literature on cell therapy using MSCs and stem cell-derived exosomes as potential treatments for SNHL. We highlight key findings and discuss the current limitations that must be addressed to advance these therapies toward clinical application.

### MSC in SNHL

Stem cells—including embryonic stem cells (ESCs), induced pluripotent stem cells (iPSCs), and MSCs—have been extensively studied in medical research for their potential to regenerate damaged tissues and organs. These cells possess the ability to self-renew and differentiate into various somatic cell types. They can also be maintained in an undifferentiated state *in vitro* for extended periods. ESCs and iPSCs have the capacity to differentiate into nearly all cell types in the body. However, the use of ESCs raises ethical concerns. iPSCs, which are generated through the genetic reprogramming of adult cells, circumvent these ethical issues. Nonetheless, both ESCs and iPSCs exhibit high genetic and epigenetic instability, as well as tumorigenic and immunogenic risks [27]. MSCs, on the other hand, are multipotent stem cells that have been isolated from nearly all organs and tissues. They possess a strong differentiation potential, primarily giving rise to mesodermal lineage cells, such as osteoblasts, chondrocytes, adipocytes, and endothelial cells. Additionally, MSCs can differentiate into non-mesodermal cells, including neuron-like cells [28]. MSCs are known for their immunomodulatory and regenerative properties and are relatively easy to cultivate and manipulate. Studies have shown that MSCs hold promise for treating damaged cochlear sensory epithelium by replacing lost HCs or neurons. Ideally, transplanted MSCs would engraft within the inner ear and differentiate into the appropriate functional cells. Several studies have explored the potential of MSC transplantation for inner ear cell regeneration, employing various experimental designs and yielding promising results (Table 1).

**Table 1:** Studies using mesenchymal stem cells for hearing restoration

**Type of MSC**	**Study model**	**Delivery site and mode: dose, timing**	**Outcome**	**Reference**
*In vitro models*				
Mouse ESC	Three ES cell lines: R1, YC5/EYFP, and ROSA26	Cell culture media: 10 days serum-free medium with N2 supplement, EGF (20 ng/mL^−1^) IGF-1 (50 ng/mL^−1^) and bFGF (10 ng/mL^−1^) for eight days	Differentiation of the embryonic stem cells into inner ear hair cell progenitors	Li, 2003 [27]
Mouse BM-MSC	Mouse BM-MSC cells	• Cell culture media: NT3 (30 ng/mL),FGF (10 ng/mL) 4–5 days followed byNT3 (30 ng/mL) and BDNF (10 ng/mlL)one week • Atoh1 transfection using lipofectamine	Development of hair cell progenitor gene profiles but not hair cell genes Expression of mature hair cell markers	Jeon, 2007 [26]
*Animal models*				
Murine neural stem cell line	Sound damaged mice and guinea pigs	Scala tympani −1.5 × 10^6^ cells in perfusion (2.5 µL/ min) 48 h after noise exposure	Significant increase of satellite cells and Type I spiral ganglion neurons in the stem cell-inject ed animals. The neural stem cells differentia ted into hair cells, supporting cells and spiral ganglion cells	Parker, 2007 [32]
Rat BM-MSC	Mouse with hearing loss induced by a mithocondrial toxin (3-nitropropionic acid-3NP)	Lateral semicircular canal −1 × 10^5^ cells, three days after 3NP	MSC observed at the site of injury ABR thresholds at 40 kHz were improved by 23%	Kamiya, 2007 [29]
Human Neural-induced BM-MSC (NI-hMSC)	Mice with neomycin induced hearing loss	Scala tympani −1 × 10^5^ cells, seven days after Neomycin	Significant increase of spiral ganglion neurons (SGN) compared to controls. Transplanted NI-hMSC expressing NeuN in the perilymphatic space, the organ of Corti, along the cochlear nerve fibers and in the spiral ganglion	Jang, 2015 [34]
Human UC-MSC	Congenital deaf albino pigs	Subarachnoid cavity: 3 × 10^5^−1 × 10^7^ cells	UC-MSC found in the stria vascularis, the basal membrane and the spiral ganglions, brain, heart, liver, kidney lung Changes of ABR waveforms	Ma, 2016 [30]
BDNF over-expressing MSC+CI	Guinea pig deafened by kanamycin and furosemid	Intracochle ar injection (2.5 × 10^5^ cells) or administration as coating of the cochlear implant (5 × 10^5^ cells)	The MSC survived for four weeks *in vivo* The alginate-MSC coating of the CI significantly prevented SGN from degeneration; MSC alone had no effect	Scheper, 2019 [37]
Mouse BM-MSC	Immunocompetent adult mouse	Intratympanic; 1 × 10^5^ cells	No oxidative stress generation, no activation of inflammation and apoptosis	Eshraghi, 2020 [28]
Mouse BM-MSC from EGFP-transgenic mice	Mouse model of cochlear fibrocytes degeneration in the spiral ligament	Posterior semicircular canal (6 × 10^5^ cells)	Regeneration or maintenance of spiral ligament (SL) fibrocytes. Improvement of endocochlear potential (EP) Moderate recovery of ABR threshold shifts	Kada, 2020 [31]
Human UC-MSC	C57BL/6 mice exposed to sound trauma	Posterior semicircular canal (1 × 10^6^ cells)	Significant rescue effect in the MSC treated animals: down-regulation of heat shock protein and cell death effectors; up-regulation of bcl-2, genes of the immune responses, cell repair and development Preservation of hair cells in the middle turn of the cochlea	Warnecke, 2021 [33]
Human ESC-derived MSC (ES-MSC)	Sprague-Dawley rats with noise-induced hearing loss	Intravenous (5 × 10^5^ cells)	The ES-MSC treated noise-exposed rats showed lower ABR thresholds at 4, 8, and 16 kHz and better preserved spiral ganglion cells and outer hair cells Reduction of cell death markers AIF, PAR, PARP, caspase 3 and cleaved caspase 3 in the ES-MSC treated rats ES-MSCs observed in the spiral ganglion area Weaker expression of Sry and STEM121 (evidencing human DNA) in the cochlea compared to the lung	Kim et al., 2022 [35]
*Human studies*				
Biohybrid cochlear electrode: (coated with autologous BM-MSC)	Humans (three patients)	Intracochlear Dose not reported	Contradictory results: one patient experienced similar speech perception in both ears, one patient had better speech perception with the biohybrid implant; the third patient showed reduced speech perception with the biohybrid implant	Roemer et al., 2016 [41]
Human autologous bone BM-MSC	Humans (two patients)	Intravenous (5 × 10^7^ cells)	No toxicities related to the treatment but also no improvement in hearing	Lee et al., 2018 [39]
UC-MSC	Children (11 children six month to six years with acquired SNHL)	Intravenous (8 to 30 × 10^7^ cells/kg body weight)	Reduction of ABR thresholds for 62.5% of patients Improved language development and myelination of white matter on MRI	Baumgartner et al., 2018 [40]

Abbreviations: MSC: Mesenchymal stem cell; ES-MSC: Embryonic stem cell-MSC; BM-MSC: Bone marrow-derived MSC; SNHL: Sensorineural hearing loss; ABR: Auditory brainstem response; NI-hMSC: Neural-induced human MSC; EGF: Epidermal growth factor; IGF: Insulin-like growth factor; BDNF: Brain-derived neurotrophic factor.

*In vitro* studies: Mouse bone marrow-derived MSCs (BM-MSCs) have been induced to differentiate into HC progenitors through the administration of growth factors. Treatment with neurotrophin-3 (NT3) and fibroblast growth factor (FGF) for 4–5 days, followed by NT3 and brain-derived growth factor (BDGF) for 7 days, led to the expression of HC progenitor markers, including Oct4, nestin, Otx2, and Musashi. Additionally, proneural transcription factors’ such as GATA3, NeuroD, Ngn1, Math1, Brn3c, and Zic2 were expressed, though mature HC markers (myosin VIIa and espin) were absent. However, transfection with Atoh1 facilitated further differentiation into mature HCs (myosin VIIa- and espin-positive) and SCs (expressing S100A, p75Trk, claudin 14, connexin 26, and Notch1) [29]. Similarly, ESCs cultured in serum-free medium with an N2 supplement differentiated into inner ear HC progenitors expressing Math1, Brn3.1, and Jagged-1, as well as mature HC markers, such as myosin VIIa, espin, parvalbumin 3, α9 acetylcholine receptor, and p27Kip1 [30].

*In vivo* studies: Mouse BM-MSCs demonstrated excellent biocompatibility after intratympanic injection in immunocompetent adult mice, with no signs of oxidative stress, inflammation, or increased apoptosis [31]. BM-MSCs isolated from rats and injected into the lateral semicircular canal of mice with hearing loss induced by 3-nitropropionic acid, a mitochondrial toxin, migrated to the injury site and were successfully visualized there. Auditory brainstem response (ABR) thresholds at 40 kHz improved by 23% [32]. Human UC-MSCs, transplanted via the subarachnoid cavity of congenitally deaf albino pigs, reached inner ear structures—including the stria vascularis, basilar membrane, and spiral ganglions—altering ABR waveforms. However, transplanted cells were also detected in the brain, heart, liver, kidney, and lungs [33]. Bone marrow stromal cells introduced into the posterior semicircular canal of mice with induced spiral ligament degeneration stimulated fibrocyte regeneration or maintenance in the spiral ligament. This intervention improved the endocochlear potential and led to a moderate recovery in ABR threshold shifts through paracrine effects [34]. Following transplantation of a neural stem cell line (cNSC) into the scala tympani of mice and guinea pigs with sound-induced damage, the stem cells were detected in the cochlea, expressing markers specific to both neural and inner ear tissues, including HCs and SCs. This differentiation may have been influenced by the cochlear microenvironment, which upregulated site-specific proteins promoting differentiation into neural, glial, HC, or SC types [35]. C57BL/6 mice exposed to sound trauma and treated with UC-MSCs exhibited significant hearing preservation. This was associated with downregulation of heat shock proteins (HSPs) and cell death effectors, along with upregulation of anti-apoptotic genes (e.g., Bcl-2), immune response genes, and those involved in cell repair and development. Histological analysis of the organ of Corti revealed preservation of HCs in the middle turn of the cochlea in treated animals [36]. Neural-induced human MSCs (NI-hMSCs) derived from bone marrow and expressing high levels of neural markers (NeuN) were transplanted into the scala tympani of mice with noise-induced hearing loss. This resulted in a significant increase in spiral ganglion neurons. NI-hMSCs were observed in the perilymphatic space, the organ of Corti, along cochlear nerve fibers, and in the spiral ganglion [37]. Adult rats with noise-induced hearing loss received intravenous injections of human ESC-derived MSCs (ES-MSCs). These animals exhibited lower ABR thresholds at 4, 8, and 16 kHz, better-preserved spiral ganglions and outer HCs, and reduced levels of HSP70 and apoptosis markers. A small number of transplanted ES-MSCs were detected in the spiral ganglion areas [38]. Cochlear implantation (CI) combined with stem cell therapy improved implant functionality [39]. CI combined with BDNF-overexpressing MSCs, delivered simultaneously into the guinea pig cochlea, reduced spiral ganglion degeneration more effectively than pre-implant BDNF treatment [40].

Clinical studies: Most clinical trials using MSCs for hearing loss are in phases I, I/II, or II [41]. A single intravenous dose of BM-MSCs administered to two adult patients with SNHL produced no toxicity but also no improvement of the hearing thresholds [42]. In another study, 11 children with acquired hearing loss received a single intravenous dose of UC-MSCs. Improvements were observed in 62.5% of patients, including reduced ABR thresholds, enhanced language development, and increased white matter myelination on MRI [43]. One clinical trial investigated biohybrid cochlear electrodes coated with autologous bone marrow-derived mononuclear cells in one ear, while the contralateral ear received a standard implant. Results varied: one patient experienced similar speech perception in both ears, another had better perception with the biohybrid implant, while a third showed reduced speech perception with the biohybrid device [44]. Although inconclusive, these findings represent early efforts to integrate stem cell therapy with cochlear implants.

Although the studies presented above reported mostly favorable outcomes—such as cochlear cell protection and lowered ABR thresholds—there are significant limitations in comparing their results due to key differences in study design. These include variations in recipient species, stem cell sources, delivery sites, dosages, treatment timing, and assessed endpoints [45]. To obtain reliable conclusions, further studies using standardized methodologiesare necessary. MSC therapy appears promising for treating various diseases that currently lack effective treatments. As of November 21, 2024, approximately 1515 trials (509 completed) involving MSCs for different conditions were registered on www.clinicaltrials.gov; however, the results so far have not justified the introduction of MSC treatments into clinical practice. Notably, there are no concluded or ongoing trials investigating MSC therapy for SNHL [46]. The use of stem cells for treating various diseases remains controversial due to concerns about potential risks, including immune rejection, limited cell survival in the host environment, and the possibility of malignant transformation [47, 48]. Moreover, producing a sufficient quantity of MSCs for clinical applications requires extensive *in vitro* expansion, which increases the risk of spontaneous transformation and genetic alterations in the cells [49].

**Figure 2.**
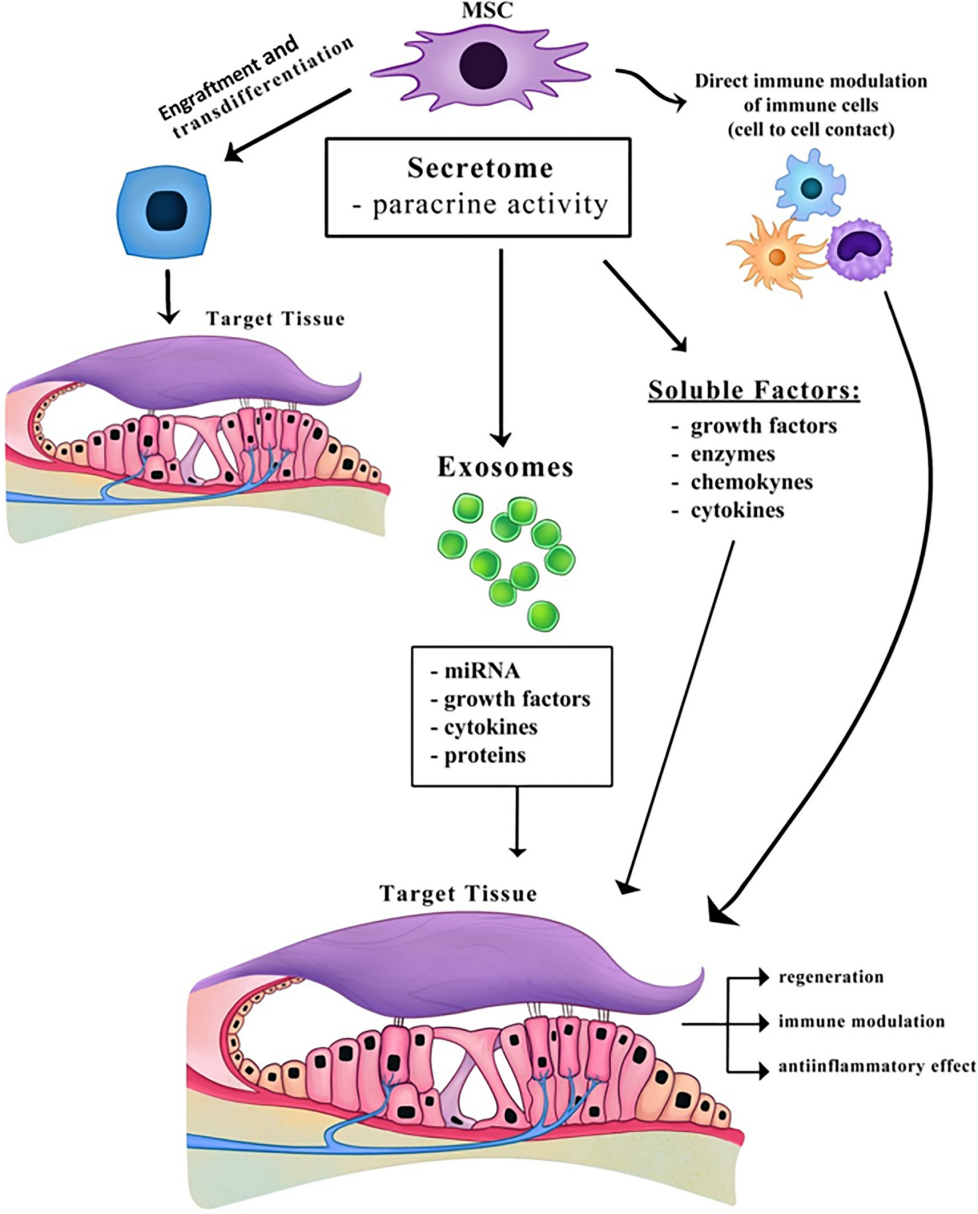
**Mesenchymal stem cells and their “secretome”—mechanism of action.** Abbreviation: MSC: Mesenchymal stem cell.

**Figure 3.**
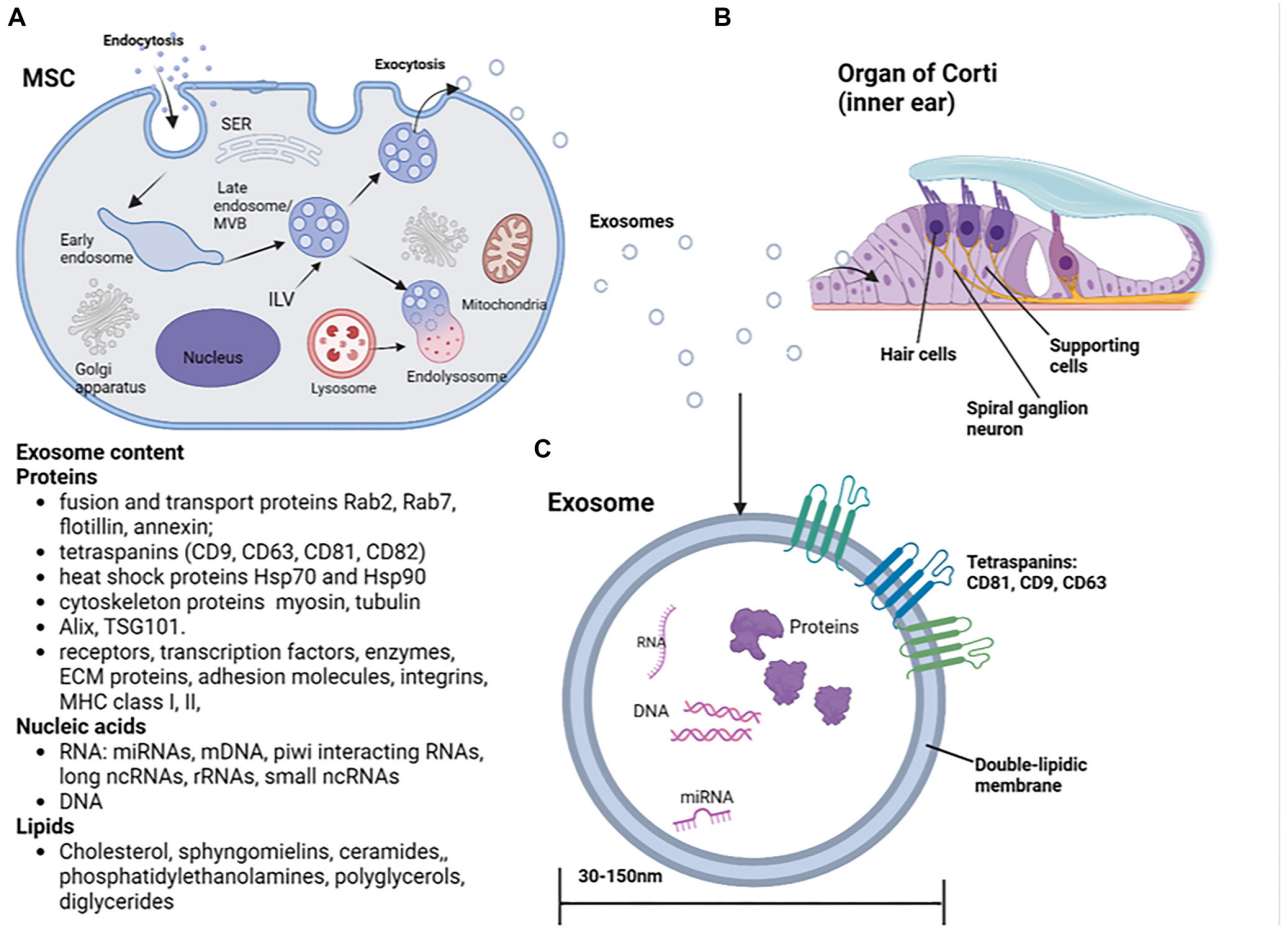
(A) The development of exosomes in the MSCs in the endosomal pathway; (B) Exosomes’ release and uptake by the target cells in the cochlea; Hair cells and supporting cells; (C) The structure and main constituents of exosomes (proteins, nucleic acids, lipids). Abbreviation: MSC: Mesenchymal stem cell.

### MSCs-derived exosomes (MSC-Exo)

MSC were initially thought to promote tissue regeneration by migrating to lesion sites, engrafting, and differentiating into mature functional cells. However, several studies suggest that MSC engraftment alone does not fully explain the extent of their regenerative effects [50]. Instead, MSC can stimulate tissue repair through alternative mechanisms, such as enhancing cellular viability, proliferation, and differentiation, remodeling the extracellular matrix, and inhibiting apoptosis, fibrosis, and inflammation. These effects are primarily mediated through paracrine signaling, involving secreted factors, such as cytokines, chemokines, hormones, and extracellular vesicles—collectively referred to as the MSC “secretome” [51] (Figure 2). Even when transplanted MSC fail to reach the inner ear, studies have shown improvements in hearing and protection of HC. For example, in a study using hhASC injected intraperitoneally into BALB/c mice with experimental autoimmune hearing loss, positive effects were observed. These benefits are attributed to the paracrine action of hASC [52]. The composition of the MSC secretome is tissue-specific, reflecting the physiological and pathological state of its tissue of origin. Notably, the secretome derived from adipose-derived stem cells is richer in bioactive factors compared to that of BM-MSC [53, 54]. Replacing direct cell transplantation with the secretome could help mitigate risks, such as unwanted differentiation, activation of an allogeneic immune response, and tumorigenicity [55]. Additionally, the secretome, as a biological therapeutic product, offers advantages including the ability to be modified for enhanced biological effects, large-scale production from commercially available cell lines, and the delivery of bioactive factors [56]. EV first gained research attention in the late 1980s [57]. These cell-derived, membrane-bound vesicles carry bioactive molecules and deliver them to recipient cells. EV are classified based on their biogenesis and size into three main categories: (1) exosomes (30–150 nm), which originate from endosomes and are generated through biogenesis, transport, and release; (2) microvesicles (100–1000 nm), which form via outward budding and shedding from the plasma membrane; and (3) apoptotic bodies (> 1000 nm), which arise during apoptosis [58, 59]. Distinguishing between exosomes and microvesicles is challenging due to their overlapping size ranges. Because no specific biomarkers uniquely differentiate them, they share similar proteins and RNAs, making classification based on molecular content difficult [60]. To address this, the International Society for Extracellular Vesicles recommends classifying EV into two broad categories: small EV (< 200 nm) and medium/large EV (> 200 nm). In the literature, the terms “exosomes” and “small extracellular vesicles” are often used interchangeably, with many authors favoring the former. In this review, we use both terms without distinction.

### Structure, composition, functions of exosomes

Exosomes are naturally produced by almost all eukaryotic cells and are transported through biological fluids [61, 62]. Their formation can be modulated by cellular stress and activation signals [63]. Exosomes originate from late endosomes (Figure 3) through the inward budding of the multivesicular body (MVB) membrane, forming intraluminal vesicles (ILVs) that incorporate specific proteins and cytosolic components. Most ILVs are released into the extracellular space when MVBs fuse with the plasma membrane, becoming extracellular vesicles known as exosomes [64]. Once released, exosomes interact with target cells and are taken up through endocytosis (e.g., phagocytosis and pinocytosis), receptor–ligand interactions, or membrane fusion [65–67]. Their uptake is cell-type specific and relies on the recognition of particular surface molecules [67, 68]. These receptor–ligand interactions can be exploited for targeted exosome delivery by modifying their surface with specific ligands that bind to target receptors [68–72]. Exosomes carry genetic and proteomic cargo that plays a crucial role in intercellular communication. Approximately 80% of the proteins found in EVs are common to all exosomes, including fusion and transport proteins (Rab2, Rab7, flotillin, annexin), tetraspanins (CD9, CD63, CD81, CD82), HSPs, cytoskeletal proteins (actin, myosin, tubulin), and proteins involved in MVB synthesis (Alix, TSG101) [73, 74]. The detection of these common proteins can be used to confirm exosome isolation [75]. Some exosomal contents are tissue-specific, including receptors, transcription factors, enzymes, extracellular matrix proteins, lipids, nucleic acids (DNA, mRNA, and miRNA), adhesion molecules (CAMs), integrins, MHC class Iand II (on B lymphocytes and dendritic cells), and transferrin receptors (on reticulocytes). The bioactive cargo of exosomes, which reflects both their cell of origin and physiological state, holds potential for identifying novel diagnostic and prognostic biomarkers [76]. Exosomes lack a nucleus and cannot replicate, but they contain abundant biologically active RNA molecules [77]. While miRNAs are the most studied, next-generation sequencing has also identified other coding and non-coding RNAs, including mitochondrial DNA, piwi-interacting RNAs, long non-coding RNAs, ribosomal RNAs, small non-coding RNAs, transfer RNAs, and circular RNAs. miRNAs regulate gene expression, while other ncRNAs, such as circular RNAs, also play active regulatory roles in recipient cells. This highlights exosomes’ role in gene regulation and their involvement in both normal development and cancer [78]. Exosomes have a bilayered lipid membrane composed of cholesterol, sphingomyelin, ceramides, and other lipids. Their lipid composition depends on their cellular origin and includes cholesterol, phospholipids, phosphatidylethanolamines, polyglycerols, and diglycerides. Compared to other EVs, exosomes have a more organized lipid structure and greater stability against detergents [79, 80]. Their membrane composition differs from the cytoplasmic membrane, contributing to exosome stability and preventing lipolytic or proteolytic degradation in circulation [81, 82]. Membrane lipids also serve as signaling mediators, interacting with prostaglandins and phospholipases C and D. Additionally, exosomes contain distinct lipid markers—higher sphingomyelin concentrations and the presence of bis(monoacylglycero)phosphate (BMP), which is exclusive to endosomes—helping to differentiate them from other EV types [83, 84]. Exosome lipid dynamics and protein domains (e.g., tetraspanin domains) play a key role in maintaining the optimal conformation of immune proteins such as MHC class II [85].

ExoCarta (http://www.exocarta.org) is a database that compiles both published and unpublished data on exosome content, serving as a valuable resource for exosome characterization. It has cataloged 9769 proteins, 3408 mRNAs, 2838 miRNAs, and 1116 lipids identified in exosomes from various cell types and organisms [86]. Exosomes play a crucial role in intercellular communication and tissue repair by transferring their contents to recipient cells through paracrine signaling. As endogenous vectors, exosomes exhibit low immunogenicity, allowing them to evade the reticuloendothelial system (RES) and avoid phagocytosis. Additionally, they can cross biological barriers, such as the blood–brain barrier (BBB) and the blood–labyrinth barrier (BLB), making them promising candidates for delivering drugs, genetic material (e.g., lncRNA, miRNA), or small molecules to otherwise inaccessible tissues, such as the brain or inner ear [87, 88]. The precise mechanisms by which exosomes influence target cells are not yet fully understood. However, certain molecular components have been identified as key mediators of specific effects. For example, miRNA-133b has been linked to recovery after ischemic stroke, while miRNA-22 has been associated with anti-apoptotic effects in cardiomyocytes during cardiac ischemia [89, 90].

### Exosome isolation methods

To obtain exosomes suitable for clinical use in SNHL, the isolation method must ensure the highest yield and purity. Differences in MSC sources, culture conditions, and EV isolation methods significantly affect the yield and quality of MSC-EV preparations [129—Witwer]. Several isolation methods have been described, each with its own advantages and disadvantages [91–103] (Table 2). To improve the efficiency of exosome isolation, different methods can be combined, such as modifying cell culture media alongside ultrafiltration and size-exclusion chromatography [104]. Selecting the appropriate separation method can be challenging and should be guided by the intended downstream applications of the exosomes [105]. A sufficient quantity must be isolated to facilitate exosome processing in tissues, making it essential to achieve both high yield and high purity [106, 107]. Ultracentrifugation (UC) is the most commonly used method for obtaining MSC-derived exosomes in clinical trials, along with tangential flow filtration (TFF) [108]. A study by Kim et al. [109] (2021) compared UC and TFF, with the latter yielding a higher concentration of exosomes isolated from human UC-MSCs. For large-scale MSC-EV production, ion exchange chromatography (IEX) and ultrafiltration (UF) have been employed. One study reported that these methods produced EV populations with significant anti-inflammatory activity in macrophages and T cells, with IEX-derived EVs exhibiting stronger effects [110].

**Table 2:** Exosome isolation methods

**Method**	**Principle**	**Advantages**	**Disadvantages**	**References**
Differential ultracentrifugation	Sequential centrifugation at high centrifugation force separation based on density and size	The gold standard of exosome isolation, suitable for large volume samples, relatively cheap high exosome yield and purity	Laborious and time consuming (more than 4 h) requires training and an expensive equipment: an ultracentrifuge	Thery et al., 2006 [89] Monguio-Tortajada et al., 2019 [90]
Size exclusion chromatography (SEC)	Based on size differences of particles. Uses the biofluid as a mobile phase and a porous gel filtration polymer as the stationary phase	High purity, short processing time (0.3 h)	Relatively low yield, can be compensated by large starting volumes	Stranska et al., 2018 [91]
Ultrafiltration	Based on the differences in size and molecular weight	Easy operation, does not request expensive equipments, high purity <4h	Loss of exosomes on filter membranes, low yield	Cheruvanky et al., 2007 [88]
Anion exchange chromatography	Based on exosome negative surface charge binding to a positively charged chromatographic matrix	High purity and reproducibility	Need additional concentration of the obtained sample by ultrafiltration	Deregibus et al., 2016 [92]
Immunoaffinity capture	Additional step to increase exosome yield and purity based on the expression of surface proteins. Uses antibodies against specific exosome surface markers (CD9, CD63, and CD81). It can use magnetic beads, nanoparticles coated with antibodies against the surface proteins, markers from parent cells, or exosome-binding molecules such as heat shock protein	Generates specific exosomes. It can isolate subsets of exosomes	Low yield, expensive, time consuming (4–20 h)	Koliha et al., 2016 [93]; Boriachek et al., 2019 [94]; Sharma et al., 2018 [95]; Ghosh et al., 2014 [96]
Precipitation with PEG based reagents	The low solubility of exosomes in the reagent leads to the formation of exosome aggregates which are then precipitate d by low-speed centrifugation	High yield, simple operation, suitable for large samples. Operation time 0.3–12 h	Low purity (potential contaminants) and specificity	Weng et al., 2016 [97]; Konoshenko et al., 2018 [98]; Li et al., 2017 [99]
Tangential flow filtration	The fluidics flow tangential to a filter membrane	High yield	Moderate purity	Busatto et al., 2018 [100]

### Exosome engineering

**Figure 4.**
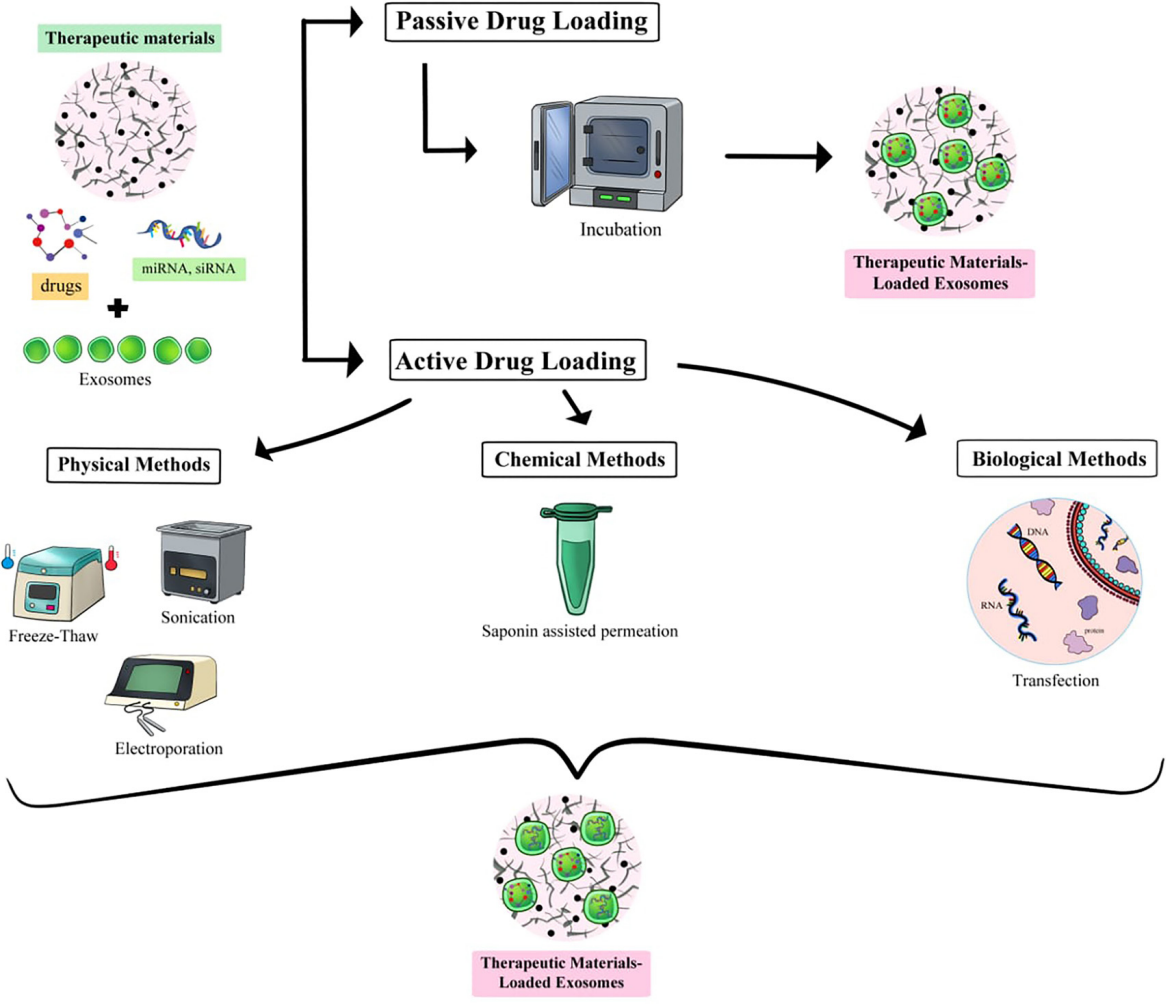
Methods of loading different cargos to target tissues through exosome engineering.

The great potential of exosomes in various pathologies has been well demonstrated, but several limitations hinder their clinical application. Naturally produced exosomes lack the ability to specifically target certain cells or tissues. These limitations can be addressed through exosome modification and the development of engineered exosomes. MSC-derived exosomes can be enriched from the vesicular “secretome” to create new therapeutic agents for various diseases, including those affecting the inner ear. Additionally, exosomes can be loaded with a range of molecules and serve as drug delivery vehicles. Engineered exosomes can be fabricated either before isolation—by manipulating the parental cells—or after isolation using chemical or mechanical methods [111]. Exosome production can be stimulated by preconditioning parental cells through hypoxia [112–114], heat shock [115], transfection, biomaterials, and other approaches [116]. Introducing exogenous drugs to donor cells allows for in situ exosome preloading. However, preloading strategies are often unsuitable for many types of cargo, necessitating *in vitro* loading of purified exosomes. Since the exosome membrane presents a barrier to cargo loading, two primary methods are used: passive loading, which involves simple incubation with therapeutic material, and active loading, which employs physical techniques such as electroporation, sonication, freeze-thaw cycles, UC, density gradients, or chemical methods like membrane permeabilization with saponin or transfection [117, 118] (Figure 4). Exosomes loaded with biopharmaceuticals exhibit improved *in vivo* stability and enhanced cell-targeting efficiency. However, several limitations remain in preconditioning and engineering methods. The chemical or physical pretreatment of MSCs cannot prevent nonspecific aggregation of exosomes during treatment [87]. Pretreatment with cytokines or chemicals may have long-term effects on the physiological properties of MSCs [81]. Engineering methods may fail to consistently produce the desired exosomes, often requiring additional modifications that increase the complexity of industrial production [118–121]. Large drug molecules may be difficult to incorporate into exosomes [122]. To overcome these challenges, close collaboration among researchers, clinicians, and regulatory authorities is essential to ensure the production of high-quality, reproducible engineered exosomes for safe application in translational medicine [123, 124].

### MSC-derived exosomes for tissue regeneration

MSCs represent an excellent source of exosomes, producing a significantly larger quantity compared to other cell lines. In pathological conditions, the paracrine gradient at the periphery of the affected organ attracts MSCs, promoting tissue healing [125]. Similar to their cells of origin, MSC-Exo possess important immunomodulatory properties, including the inhibition of mitogen-activated T cells, the induction of an anti-inflammatory phenotype in naïve dendritic cells and NK cells, and the suppression of B cells. They contribute to maintaining tissue homeostasis, play a crucial role in intercellular communication, and can restore normal tissue function through active catalytic enzymes [126–128]. The composition of exosomes is specific to their tissue of origin. Baglio et al. (2015) compared the small RNA profiles of exosomes released by ASC and BM-MSC using RNA sequencing. Their study found that the two types of exosomes contained different tRNA species, which could have significant implications for clinical applications [129]. When comparing the ability of small EVs and conditioned medium (with equivalent protein concentrations) to induce de novo adipose cell regeneration, EVs showed superior performance, with the only advantage of conditioned medium being its greater availability [130]. Currently, 25 ongoing or completed clinical trials involve exosomes, with the majority utilizing MSC-derived exosomes [131]. However, their use as therapeutic agents remains challenging, particularly when primary MSCs serve as the cellular source of exosomes, due to their inherent heterogeneity. Multiple factors contribute to this heterogeneity, including the tissue of origin, donor profile variations, isolation methods, and culture conditions. Additionally, production process parameters can influence the characteristics of exosome products, highlighting the need for stringent quality control assays before their application in clinical trials [132, 133]. Exosomes released from stem cells have the potential to exert the same therapeutic and clinical benefits as the cells themselves. They can promote tissue regeneration and repair damaged tissues, such as in myocardial infarction [134–136] or cisplatin-induced renal injury [137]. UC-MSC-derived exosomes have demonstrated anti-inflammatory effects by reducing tumor necrosis factor-α (TNF-α) and interleukin-1 (IL-1) expression while increasing neuronal growth factors [138]. However, it is essential to consider that exosome administration can also lead to adverse effects depending on their origin, emphasizing the importance of rigorous safety testing [139].

**Table 3:** Studies using exosomes isolated from MSC for SNHL

**Exosome origin**	**Recipient species**	**Delivery site and mode: dose, timing**	**Outcome**	**Reference**
Human UC-MSC	BV-2 Microglial cell line activated with lipopolysaccharide (LPS)	1.2 × 10^8^ exosomes/mL in the culture medium, 1 h before LPS	Anti-inflammatory effect Significant reduction of IL-1β gene expression; phosphorylation level of NF-κ β p65 was significantly diminished	Warnecke et al., 2020 [137]
	Primary rat SGN cell culture	UC-MSC-EVs from 1 × 10^6^, 2 × 10^6^, and 4 × 10^6^ cells in the culture medium	Improved survival, increased primary neurite growth dose-dependently	
	One-month old female C57BL/6 mice exposed to noise	Posterior semicircular canal 72 h after noise trauma 1 µL EV (2 × 10^10^ particles/mL)	Five days after delivery Protection of the inner ear cells, partial hearing restoration: reduced ABR thresholds; rescue of the organ of Corti	
Human UC-MSC (Wharton’s jelly)	Mice with intraperitoneal Cisplatin induced hearing loss	100 µL of UC-MSC exosomes (1.2 µg/µL) intraperitoneal injection and 10 µL UC-MSC exosomes through the round window niche (RWN)	Significant reduction of ABR threshold of 8 and 12 kHz; rescue of the lost cochlear hair cells; reversed miRNA profile of the cochlear tissue	Tsai et al., 2021 [138]
Human UC-MSC	Human subject with bilateral hearing loss (Meniere disease)	Intracochlear, simultaneously with cochlear implant −1 × 10^8^ particles/µL	No toxicity Better speech intelligibility Significantly higher mean impedances in the EV-treated side	Warnecke et al., 2021 [139]
Human BM-MSC	Cochlear explants from ICR mice treated with Cisplatin and co-cultured with MSC	Exosomes isolated from the culture medium of the co-culture of MSC with cochlear explants 2.48 × 10^10^ particles/mL diluted to 1-, 3- or 5-fold; 24 h before Cisplatin	Enrichment of HSP70 in the secreted exosomes Reduced Cisplatin induced ototoxicity Decreased hair cell death	Park et al., 2021 [140]
Heat shock treated mouse BM-MSC	C57BL/6 mice treated with intraperitoneal Cisplatin	1.2 µg/µL, 1 µL trans Tympanic 30 min after Cisplatin	Exosomes reduced Cisplatin ototoxicity Diminished ABR thresholds; reduced hair cell loss, reduced inflammation, decreased apoptosis	Yang et al., 2022 [141]
Mouse inner ear stem cells	*In vitro*: HEI-OC1 cells exposed to Gentamycin	Culture medium: 0, 0.01, 0.1, and 0.3mg/mL same time as Gentamicin	Improved cell viability Reduced oxidative stress Increased relative miR-182-5p expression and decreased FOXO3	Lai et al., 2020 [143]
Mouse cochlear spiral ganglion progenitor cells	Female C57BL/6 mice ischemia-reperfusion injury (I/R) model of hearing loss	Intracochlear: 0.1 µg, 0.2 µg, 0.5 µg, and 1 µg/1 µL, 1 h before the ischemia-reperfusion injury and every 12 h after the injury	Significant ly decreased the threshold shift at 8, 16, 32 kHZ Prevented hair cell damage Anti-inflammatory effect: IL-6, IL-1β, TNF-α, and Cox-2, were significantly reduced Inhibition of hair cells apoptosis	Yang et al., 2021 [144]
UC-MSC (Promocell)	Hei-OC1 cell line treated with Neomycin	30 µg/mL for 24 h in the cell culture medium, 24 h after Neomycin	Exosomes reduced hearing and hair cell loss caused by neomycin; modulated autophagy in hair cells, upregulated endocytic gene expression; promoted cell survival, decreased oxidative stress and apoptosis in hair cells	Liu et al., 2024 [145]
	Cochlear explants treated with Neomycin	30 µg/mL for 24 h in the cell culture medium, 24 h after Neomycin		
	C57BL/6 mice deafened by Neomycin	Round window niche (RWN): 20 µg in 10 µlLPBS) two days after Neomycin. ABR, immune staining after two weeks	Exosomes attenuated hearing loss (lower ABR thresholds) and reduced the loss of Myo 7a-positive hair cells in the middle and basal regions of the cochlear tissues	
Rat BM-MSC	Spiral ganglion culture treated with OuabainSD rats deafened by intratimpanic Ouabain	2 µg/µL in cell culture media 48 h after Ouabain 200 µg/mL, together with 20 mM ouabain. Seven days after treatment: ABR, immunostaining	Significant increase of neurite growth and growth cone development Prevent SGN degeneration EV rescued ouabain-induced hearing loss rescuing the threshold shifts induced by ouabain EV Protected SGN from degeneration Inhibit ouabain Induced apoptosis	Chen et al., 2024 [146]

Abbreviations: MSC: Mesenchymal stem cell; BM-MSC: Bone marrow-derived MSC; SNHL: Sensorineural hearing loss; ABR: Auditory brainstem response.

### MSC derived exosomes in SNHL therapy

There are few studies using exosomes to treat SNHL, but the results are encouraging. The proliferative and protective factors specific to MSC-derived exosomes help safeguard inner ear sensory cells from ototoxic injuries while stimulating cellular and tissue regeneration (Table 3). In one study, human UC-MSC-Exo improved survival and primary neurite growth in rats, reduced HC loss, and partially restored hearing—demonstrating both neuroprotective and regenerative effects. Gene panel analyses further revealed that UC-MSC-Exo modulated the expression of multiple genes involved in tissue remodeling and repair [140, 141]. In an experimental study investigating ways to reduce CI-related inflammation, a subject who had received a CI in one ear underwent implantation in the contralateral ear four years later, this time with intracochlear UC-MSC EVs. After 24 months, speech intelligibility improved, and mean impedances on the EV-treated side were significantly higher [142]. Exosomes enriched in HSP70—either produced by heat-shock-preconditioned BM-MSCs or by direct exosome treatment—reduced cisplatin-induced ototoxicity in cochlear explants. These exosomes lowered levels of the pro-inflammatory cytokines IL-1, IL-6, and TNF-α while increasing the anti-inflammatory cytokine IL-10 in mice [143, 144]. Similarly, hypoxia-preconditioned BM-MSC-derived exosomes, which overexpress HIF-1, limited HC loss and inhibited oxidative stress caused by cisplatin in mice [145]. Exosomes derived from inner ear tissues have also shown otoprotective effects. For instance, exosomes from inner ear stem cells prevented gentamicin-induced ototoxicity [146], while those from cochlear spiral ganglion progenitor cells inhibited inflammation and attenuated ischemia-reperfusion-induced cochlear damage [147]. UC-MSC-derived exosomes added to the HEI-OC1 cell line and cochlear explants following neomycin exposure reduced HC loss, modulated autophagy, upregulated endocytic gene expression, promoted cell survival, and decreased oxidative stress and apoptosis. In mice deafened by neomycin, these exosomes reduced hearing loss [148]. Additionally, BM-MSC-derived EVs increased neurite growth, enhanced growth cone development, and prevented SGN degeneration after ouabain exposure. *In vivo*, they rescued ouabain-induced hearing loss by protecting against SGN degeneration [149]. Analyzing these findings, it is evident that MSC-derived exosomes—regardless of tissue origin or recipient species—consistently protect inner ear tissues from ototoxic damage while promoting regeneration. However, no clinical trials using MSC-derived exosomes for SNHL have been recorded to date, as current data remain insufficient and unreliable. A major limitation of existing studies is the heterogeneity of study designs, including variations in MSC sources, exosome isolation methods, characterization techniques, exosome dosing (expressed as micrograms of protein or particle numbers), application schedules, administration sites, incubation times, and evaluated endpoints.

## Conclusion

Technological breakthroughs are providing new and promising tools for managing hearing loss. While inner ear HC regeneration remains challenging, it has been proven possible. Exosomes produced by MSCs present new opportunities in regenerative medicine. However, high-quality clinical trials are necessary to evaluate their future use in treating SNHL. Exosomes offer several advantages over MSC-based cell therapy. Since they cannot replicate, they are not tumorigenic, and their use does not raise ethical concerns. They also lack immunogenicity, and their small size allows them to cross natural barriers, making them effective carriers for drugs, genetic material, or small molecules. Additionally, exosomes are stable and can be stored long-term. Advances in exosome engineering enable modifications to enhance their contents and surface markers, improving targeted delivery and therapeutic efficacy. Despite these advantages, several challenges hinder their clinical application. Batch-to-batch variations arise due to differences in donor cell status and isolation methods. Additionally, large quantities of exosomes are required, and regulatory frameworks are still lacking. To move forward with clinical trials, exosome production must be optimized and standardized through automated manufacturing processes that enable large-scale production and quality control. Another critical concern is safety. Potential off-target effects and long-term risks must be thoroughly assessed. Addressing these challenges will require collaborative efforts from scientists, biotechnology companies, and regulatory authorities.
